# Analysis of the Prognosis Outcomes and Treatment Delay Among ST-Segment Elevation Myocardial Infarction Patients in Emergency Department Based on the Presence of Symptoms Suggestive of COVID-19

**DOI:** 10.34172/ijhpm.2024.8207

**Published:** 2024-05-18

**Authors:** David Samuel Kwak, Joonbum Park

**Affiliations:** ^1^Department of Family Medicine, Soonchunhyang University Seoul Hospital, Seoul, Republic of Korea.; ^2^Department of Emergency Medicine, Soonchunhyang University Seoul Hospital, Seoul, Republic of Korea.

**Keywords:** Emergency Service, COVID-19, Patient Safety, Myocardial Infarction, Isolation, Korea

## Abstract

**Background:** During COVID-19 pandemic, the emergency department (ED) was challenged to treat patients with COVID-19-related symptom. Therefore, the aim of this study was to investigate treatment delay and prognostic outcomes in ST-segment elevation myocardial infarction (STEMI) patients during COVID-19 pandemic due to isolation or precaution and compare it with pre-COVID-19 period.

**Methods:** This was a retrospective observation study using multicenter data with different case mix. Anonymized data were collected through each center’s electronic medical data of common case report form. Primary outcomes were number and rate of in-hospital mortality within 28 days. Secondary outcomes were door-to-balloon time and length of stay in the ED. Kaplan-Meier estimation and Cox proportional hazard regression analysis were performed to determine impact of predictors on 28-day in-hospital mortality.

**Results:** Door-to-balloon time was longer in STEMI patients with COVID-19-related symptom(s) than those without symptom during the COVID-19 period (97.0 [74.8, 139.8] vs. 69.0 [55.0, 102.0] minutes, *P*<.001). However, there was no significant statistical difference in door-to-balloon time between STEMI patients with and without COVID-19-related symptom(s) during the pre-COVID-19 period (73.0 [61.0, 92.0] vs. 67.0 [54.5, 80.0] minutes, *P*=.2869). The 28-day mortality rate did not show a statistically significant difference depending on symptoms suggestive of COVID-19 during the pre-COVID-19 period (15.4% vs. 6.8%, *P*=.1257). However, it was significantly higher during the COVID-19 period (21.1% vs. 6.7%, *P*=.0102) in patients with COVID-19 suggestive symptoms than in patients without the symptoms.

**Conclusion:** In Korea, symptoms suggestive of COVID-19 during the pandemic had a significant effect on the increase of door-to-balloon time and 28-day mortality in STEMI patients. Thus, health authorities need to make careful decision in designating symptoms indicated for isolation in ED based on opinions of various medical field experts.

## Background

**Key Messages**

**Implications for policy makers**

- As patients are more likely to be reluctant to visit the emergency room during the worldwide pandemic, public campaigns should be strengthened for informing the symptoms implicative of time-dependent diseases.
- The parameter of symptoms (such as fever and dyspnea) that the government designates to be treated in isolation room in pandemic situations has a significant impact on emergency department (ED) treatment process.
- If acute myocardial infarction (AMI) patients have symptoms suggestive of COVID-19, treatment could be delayed in ED leading to an increase of 28-day mortality rate.
- Solutions for delayed ED treatment process of AMI patients in the pandemic period are needed.

**Implications for the public**

During the COVID-19 pandemic, the emergency department (ED) faced challenges in treating acute patients exhibiting symptoms suggestive of COVID-19 due to the mandate for isolation or precautionary measures. If critically ill emergency patients visiting the ED had concurrent respiratory infectious diseases requiring isolation, the delay in timely treatment due to precautionary measures could lead to dire consequences in the patient’s prognosis. Acute myocardial infarction (AMI) patients with COVID-19-related symptom(s) were delayed in treatment with a higher 28-day mortality rate than those without symptom. This study should provide grounds upon which the government and clinicians should strive to define the most appropriate isolation-requiring symptoms to minimize the spread of pandemic disease without increasing the mortality rate of time-dependent disease such as AMI.

With COVID-19, healthcare systems around the world are facing new challenges. In Korea, such respiratory-related infectious diseases occur nationwide roughly every five years.^1-3^

 Emergency rooms serve as the initial point of entry for patients presenting symptoms of upper respiratory infectious diseases. They are required to facilitate significantly greater amount of equipment and manpower in treating patients suspected of a pandemic disease. Therefore, as the number of suspected infectious patients increase during the pandemic, emergency department (ED) and hospital resources become strained, affecting overall ED treatment capacity.^4-6^

 In the case of time-dependent diseases such as ST-segment elevation myocardial infarction (STEMI) and acute ischemic stroke, this effect might result in missing the appropriate timing for treatment.

 COVID-19 pandemic affects healthcare globally. Although its impact is somewhat decreasing, it continues to this day. During the COVID-19 period, healthcare resources have been heavily concentrated in treating COVID-19 patients.^7,8^ Many hospitals have been transformed into facilities for COVID-19 patients, which has reduced access for patients with other diseases to the hospitals. There is a great risk that can stem from delay made in diagnosis and treatment for acute patients with time-dependent diseases such as STEMI.^9,10^ But the combination of decentralization of medical resources, lockdown regulations, social distancing guidelines, and public fear of COVID-19 infection has also made visiting hospitals more difficult.^11-14^

 Several studies have demonstrated that numbers of percutaneous coronary intervention (PCI) procedures given to STEMI patients have decreased.^15-17^ It has already been proven worldwide that delayed treatment of STEMI increases mortality.^18-21^

 There were even cases in which emergency room treatment was delayed until COVID-19 was confirmed negative, to the patients presenting the suspected symptoms based on Centers for Disease Control and Prevention guidelines.^22^

 There has been no study analyzing the effect of COVID-19-related symptom on delay of procedure in ED during COVID-19 period compared with that during the pre-COVID-19 period. Thus, we aimed to determine whether STEMI treatment was delayed by the presence of symptoms suggestive of COVID-19 in the emergency room, and to discern the effects of such delay in the STEMI patients during the COVID-19 pandemic period.

 We analyzed and compared patients with and without symptoms suggestive of COVID-19 during the pre-COVID-19 period and the COVID-19 period and examined whether having the symptom influenced the treatment process and prognosis in STEMI patients.

## Methods

###  Study Design and Population

 This was a retrospective observation study of multicenter data with different case mix. The three hospitals participating in this study exhibit regional differences as they are in metropolitan, urban, and suburban areas. Additionally, there are variations among the hospitals in terms of patient demographics, including primary symptoms, severity, and age groups. Each emergency medical center was visited by 1 to 15 STEMI patients a month. The inclusion period was 1 year of the COVID-19 period (from May 1, 2020 to April 30, 2021). Data were compared with those retrospectively collected in the same duration window during the pre-COVID-19 period from May 1, 2018 to April 30, 2019.

###  Inclusion Criteria

 STEMI and non-STEMI must be diagnosed in the emergency room to determine the subsequent treatment process. In this study, diagnosis in ED between STEMI and non-STEMI are confirmed by a cardiologist. This study aimed to analyze delays in the diagnosis and treatment process for STEMI patients in the emergency room. Therefore, STEMI and non-STEMI were defined based on the emergency room diagnosis rather than the final diagnosis.

###  Data Collection

 Anonymized data were collected through each center’s electronic medical data of common case report form. Each center identified a local principal data supervisor. We collected demographic, clinical, and procedural data, including door-to-electrocardiogram (EKG) time, door-to-balloon time, COVID-19-related symptom(s) in ED (defined by national COVID-19 guideline; fever, dyspnea, cough/sputum/chill), PCI procedural data, and in-hospital morality. After each participating center submitted data filled form to Soonchunhyang University Seoul Hospital in charge of data analysis, data were collected and analyzed.

###  Study Outcomes

 Primary outcomes were number and rate of in-hospital mortality within 28 days for STEMI patients (excluding those who died from complications other than STEMI, such as pneumonia). Secondary outcomes in a study included both the door-to-balloon time and the length of ED stay. Door-to-balloon time is defined as the duration from a patient’s arrival at the ED to the moment when the catheter guidewire reaches the culprit lesion and balloon angioplasty is performed. Additionally, length of stay is defined as the duration from the time the patient arrives in the emergency room until the departure from the emergency room to move to the inpatient room. When analyzing 28-day mortality, patients who died from causes other than AMI, such as pneumonia, were excluded.

###  Statistical Analysis

 All statistical analyses were performed using Rex Excel-based statistical analysis software version 3.6.0 (RexSoft, Korea, http://rexsoft.org/) based on R version 4.0.0 (R foundation for Statistical Computing, Austria). Quantitative variables were described using median and interquartile range. Absolute frequency and percentage were used for categorical variables. Mann-Whitney U test and chi-square test were used for continuous and categorical variables, respectively. Paired sample signed rank test was used for paired continuous variables. Normal distribution of continuous variables was tested by the Shapiro-Wilk test. Kaplan-Meier estimation and Cox proportional hazard regression analysis were performed to identify the impact of predictors on 28-day in-hospital mortality.

## Results

###  Overview of Collected Data

**Figure 1:**
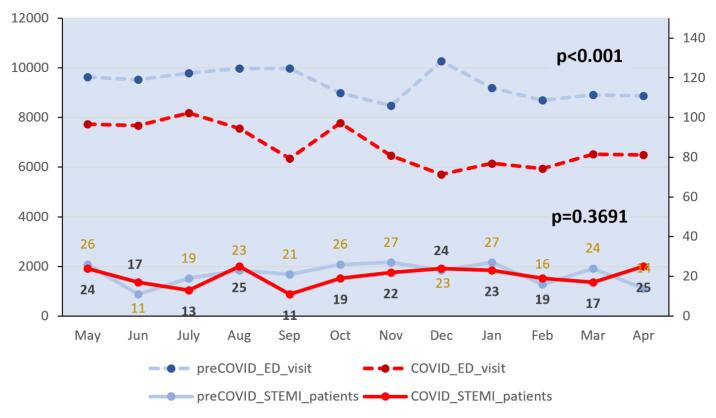
Reduction of Emergency Department Visits in Comparisons of the Pre-COVID-19 (Median: 9354.0, IQR: 8902.0-9831.3) and the COVID-19 Period (Median: 6512.0; IQR: 6303.3, 7693.3, *P* < .001), Showing No Significant Difference in the Number of ST-Segment Elevation Myocardial Infarction Patients Between Pre-COVID-19 (Median: 23.0, IQR: 18.25, 26.00) and COVID-19 Period (Median: 20.5, IQR: 17.00, 24.00, *P* = .3691). Abbreviations: ED, emergency department; STEMI, ST-segment elevation myocardial infarction; IQR, interquartile range.

 A total of 995 patients with acute myocardial infarction (AMI) were analyzed in this study, including a total of 508 patients with STEMI and 487 patients with non-STEMI. None of these study subjects was confirmed with COVID. The median monthly total number of patients visiting the three hospitals in the year prior to COVID-19 was 9354. It decreased significantly to 6512 during the COVID-19 period (*P* < .001). However, the median monthly number of STEMI patients showed no statistically significant difference: 23 (19.25, 26.25) before COVID-19 vs. 22 (17.00, 24.00) during COVID-19 period (*P* = .3243, Figure 1).

**Figure 2:**
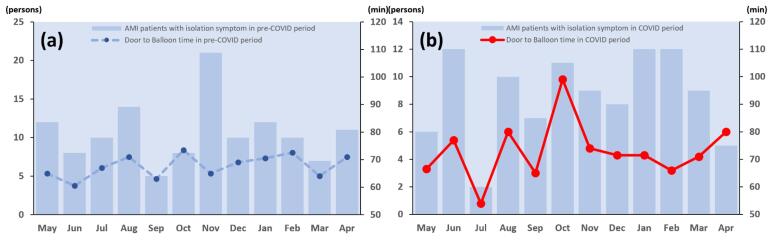
Monthly Median Values of Door-to-Balloon Time With the Numbers of Acute Myocardial Infarction Patients With Symptoms Suggestive of COVID-19 During (a) Pre-COVID-19 Period (Pearson Correlation = -0.0901, *P* = .7807) and (b) COVID-19 Period (Pearson Correlation = 0.6728, *P* = .0165). Abbreviation: AMI, acute myocardial infarction.

 There was no statistically significant correlation of door-to-balloon time with symptoms suggestive of COVID-19 among monthly AMI patients visiting the emergency room during the pre-COVID-19 period (Pearson correlation value = -0.0901, *P* = .7807). However, there was a significant correlation during the COVID-19 period (Pearson correlation value = 0.6728, *P* = .0165, Figure 2). This means that AMI patients with COVID-19 suggestive symptoms during the COVID-19 period might have been delayed in door-to-balloon time which is a critical duration for definitive treatment required for STEMI patients.

###  Baseline Demographic and Clinical Characteristics

 The higher proportion of patients 65 years of age or older within the category showing symptoms suggestive of COVID-19 (*P* = .0219) during pre-COVID-19 period was analyzed to be statistically significant while the similar proportion during COVID-19 period was not statistically significant (*P* = .1236). There were significant statistical differences of vital signs between patients with the symptoms and those without such symptoms except mean arterial pressure in each group. In intervention category, PCI treatment rate (*P* < .001) showed significant difference during the pre-COVID-19 period between those having the symptoms and who did not. Particularly noteworthy is that during the pre-COVID-19 period, the proportion of STEMI patients who did not undergo PCI was 20%, totaling 6 cases. Among these, 2 cases resulted in patient mortality prior to PCI, and in 4 cases, guardians refused CAG and PCI based on patient condition. Door-to-balloon time was longer for patients with COVID-19-related symptom(s) than that for those without the symptom during the COVID-19 period (97.0 [74.8, 139.8] vs. 69.0 [55.0, 102.0] minutes, *P* < .001) (Table 1). Furthermore, we compared the characteristics between the pre-COVID-19 and COVID-19 periods correlated to the presence of symptoms suggestive of COVID-19. In STEMI patients who exhibited COVID-19 symptoms, a prolonged door-to-balloon time was observed during the COVID-19 period. However, in patients without the symptoms, there was no statistically significant difference in door-to-balloon time (Supplementary file 1, Table S1).

**Table 1:** Characteristic of ST-Segment Elevation Myocardial Infarction Patients Who Visited Emergency Departments During Pre-COVID-19 and COVID-19 Periods According to The Presence of Symptoms Suggestive of COVID-19

**Myocardial Infarction**	**Pre-COVID-19 (n = 257)**		***P*** ** Value**	**COVID-19 (n = 239)**		***P*** ** Value**
	**Symptoms Suggestive of COVID-19**			**Symptoms Suggestive of COVID-19**		
	**Yes (n = 31)**	**No (n = 226)**		**Yes (n = 39)**	**No (n = 200)**	
Age			.0219			.1236
18-64	13 (41.9%)	147 (65.0%)		16 (41.0%)	112 (56.0%)	
≥65	18 (58.1%)	79 (35.0%)		23 (59.0%)	88 (44.0%)	
Gender			.0128			.0102
Male	21 (67.7%)	197 (87.2%)		25 (64.1%)	167 (83.5%)	
Female	10 (32.3%)	29 (12.8%)		14 (35.9%)	33 (16.5%)	
COVID-19-related symptom						
Fever	8 (25.8%)	-		2 (5.1%)	-	
Dyspnea	19 (61.3%)	-		30 (76.9%)	-	
Other URI symptoms	4 (12.9%)	-		7 (18.0%)	-	
Vital signs						
Mean arterial pressure (mm Hg)	93.3 (81.0, 109.2)	96.7 (83.3, 113.3)	.4353	93.3 (80.0, 112.8)	98.5 (85.8, 113.3)	.3982
Heart rate (/min)	97.0 (87.0, 110.5)	76.0 (65.0, 89.0)	<.001	89.0 (77.0, 100.5)	78.5 (66.0, 93.0)	.0128
Respiratory rate (/min)	20.0 (18.0, 23.0)	18.0 (18.0, 20.0)	.0018	20.0 (20.0, 26.0)	18.0 (18.0, 20.0)	<.001
Body temperature (°C)	36.8 (36.1, 37.4)	36.3 (36.0, 36.7)	.0035	36.5 (36.0, 36.9)	36.2 (35.8, 36.5)	.0401
SpO2 (%)	95.0 (87.0, 97.5)	98.0 (94.0, 99.0)	.0088	95.0 (85.0, 98.0)	97.0 (97.0, 99.0)	.5021
Interventions						
PCI treatment	25 (80.7%)	222 (98.2%)	<.001	34 (87.2%)	186 (93.0%)	.2080
Door-EKG time (min)	8.0 (4.5, 11.5)	6.0 (3.0, 9.0)	.0616	7.0 (5.0, 16.0)	8.0 (4.0, 12.0)	.3919
Door-to-balloon time (min)	73.0 (61.0, 92.0)	67.0 (54.5, 80.0)	.2869	97.0 (74.8, 139.8)	69.0 (55.0, 102.0)	<.001

Abbreviations: URI, upper respiratory syndrome; SpO_2_, oxygen saturation of peripheral capillary; PCI, percutaneous coronary intervention; EKG, electrocardiogram.

###  Procedural Characteristics and In-hospital Clinical Outcomes of STEMI Patients

 The 28-day mortality rate did not show a statistically significant difference during the pre-COVID-19 period between those who had symptoms suggestive of COVID-19 and those who did not (15.4% vs. 6.8%, *P* = .1257). However, during the COVID-19 period, the 28-day mortality rate was higher in the patients with the symptoms than without the symptom(s) (21.1% vs. 6.7%, *P* = .0102) (Table 2). When comparing procedural characteristics and clinical outcomes between pre-COVID-19 and COVID-19 periods in patients with the symptoms, no statistically significant difference was observed between the two periods (Supplementary file 1, Table S2).

**Table 2:** Comparing Outcomes of ST Segment Elevated Myocardial Infarction Patients Who Visited Emergency Departments During Pre-COVID-19 and COVID-19 Periods

	**Pre-COVID-19 (n = 257)**		***P*** ** Value**	**COVID-19 (n = 239)**		***P*** ** Value**
	**Symptoms Suggestive of COVID-19**			**Symptoms Suggestive of COVID-19**		
	**Yes (n = 31)**	**No (n = 226)**		**Yes (n = 39)**	**No (n = 200)**	
Length of ED stay	144.0 (76.5, 269.0)	108.0 (66.0, 163.0)	.1259	117.0 (80.0, 217.0)	139.5 (83.5, 292.8)	.5569
ICU admission or Death in ED	25 (80.7%)	214 (94.7%)	.0123	32 (82.1%)	174 (87.0%)	.5715
Admission days	8.0 (3.0, 13.0)	5.0 (3.0, 7.0)	.2261	6.0 (3.0, 9.5)	4.0 (3.0, 7.0)	.2700
Mortality within 28 days	4 (15.4%)	15 (6.8%)	.1257	8 (21.1%)	13 (6.7%)	.0102

Abbreviations: ED, emergency department; ICU, intensive care unit.

###  In-hospital Clinical Outcomes of Non-STEMI Patients

 For NSTEMI patients, there were no items showing differences between pre-COVID-19 and COVID-19 periods. Door to EKG time and admission days were both longer in patients with symptoms suggestive of COVID-19. Remaining items were found to have no statistically significant differences. For NSTEMI patients, there was no statistical difference in major outcome regardless of the period (COVID-19 period or not) (Supplementary file 1, Table S3).

**Figure 3:**
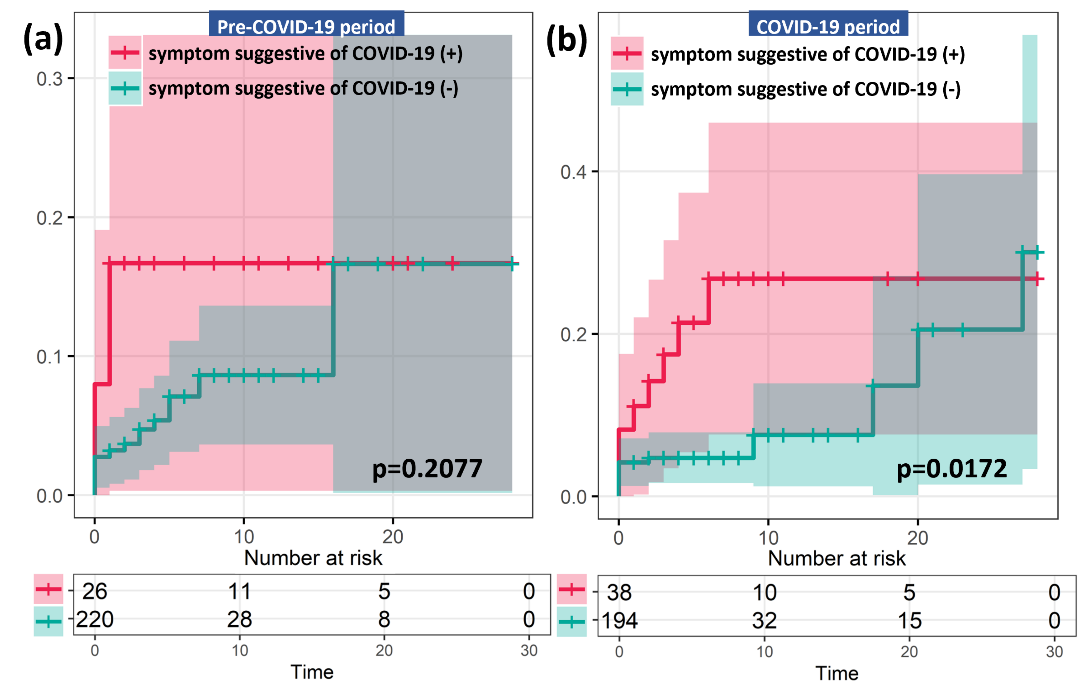
Cumulative Hazard Ratio of 28-Day Mortality According to The Presence of Symptoms Suggestive of COVID-19 During (a) Pre-COVID-19 and (b) COVID-19 Periods.

 The cumulative hazard ratio of the 28-day mortality rate for STEMI patients with the COVID-19 suggestive symptoms during the pre-COVID-19 period was 2.5076 compared with patients without the symptoms (*P* = .2077). However, the cumulative hazard ratio was higher at 2.7603 during the COVID-19 period for STEMI patients with the symptoms than for those without (*P* = .0172, Figure 3).

 We performed Cox proportional hazard regression analysis to determine factors affecting the risk of the 28-day mortality. During the pre-COVID-19 period, 28-day mortality was high in patients with an abnormal saturation pulse oxygen (SPO_2_). During the COVID-19 period, there were no statistically significant factors influencing the 28-day mortality rate. However, in contrast to the pre-COVID-19 period, factors such as the presence of symptoms suggestive of COVID-19, and the door-to-balloon time, during the COVID-19 period, emerged meaningful in simple regression, and were thus included in the Cox proportional hazard regression analysis (Table 3).

**Table 3:** COX Proportional Hazard Regression for Predictors of 28-Day Mortality in ST-Segment Elevation Myocardial Infarction Patients Who Visited Emergency Department During Pre-COVID-19 and COVID-19 Periods

**Predictors**	**Pre-COVID-19 Period**			**COVID-19 Period**		
	**Estimate**	**Hazard Ratio (95% CI)**	***P*** ** Value**	**Estimate**	**Hazard Ratio (95% CI)**	***P*** ** Value**
Symptom suggestive of COVID-19 (+)				1.5444	4.6851 (0.9750, 22.5142)	.0538
MAP (<70, >100 mm Hg)	0.6903	1.9943 (0.5312, 7.4871)	.3065			
HR (<50 or >100 beats/min)	0.8362	2.3075 (0.5837, 9.1221)	.2331			
SpO2 (<90%)	1.6837	5.3857 (1.1223, 25.8437)	.0354	1.2050	3.3366 (0.6558, 16.9770)	.1466
Not-alert mentation	0.6863	1.9864 (0.4127, 9.5605)	.3919	1.3880	4.0070 (0.6836, 23.4877)	.1240
Door-to-balloon time				-0.0001	0.9999 (0.9947, 1.0051)	.9616

Abbreviations: CI, confidence interval; HR, heart rate; MAP, mean arterial pressure; SPO_2_, oxygen saturation of peripheral capillary.

## Discussion

 This study attempted to examine not the effect of COVID-19 on STEMI from the disease itself, but the effect from, if there was, the delay in treatment of emergency patients due to changes in treatment process caused by respiratory pandemic. None of the STEMI or non-STEMI patients included in our study had COVID-19. Therefore, the pathological effect of COVID-19 on heart disease did not affect our study.

 Although several studies have shown that the number of STEMI patients and PCI procedures has decreased,^15,23-25^ our study showed that despite the decrease in the total number of emergency room patients, the number of STEMI patients and PCI procedures were not significantly different from those during the pre-COVID-19 period.

 This was likely due to the characteristics of Korea providing an easier medical accessibility with relatively abundant medical resources in a small geographical territory. Therefore, the situation in Korea is different from that in studies conducted in other countries where STEMI patients’ hesitation to visit the emergency room, social distancing, and local lockdown decreased the number PCI of STEMI patients.^15,23-25^

 Many studies on the relationship between COVID-19 and STEMI have compared characteristics of STEMI patients between COVID-19 and pre-COVID-19 periods.^26,27^

 However, our study analyzed the effects from the changes made in the process of treatment (such as having to be treated in isolation rooms, with only 2 to 4 availabilities per hospital) and in the preparation process required to protect medical staff from infections during COVID-19 period, on prognosis of STEMI patients. To the best of our knowledge, no research has been conducted on the treatment process for emergency room STEMI patients in relation to the presence of symptoms suggestive of COVID-19. This study is the first research done regarding changes (namely isolation) made in the care process for patients in the emergency room during a respiratory pandemic.

 In comparisons of treatment processes and outcomes, no difference was found in the lengths of stays in ED between the two periods. Interestingly, during the COVID-19 period, the length of the stay among patients with the symptoms was shorter than that of patients without the symptoms (117.0 [80.0, 217.0] vs. 139.5 [83.5, 292.8] minutes, *P* = .5569).This was a result different than expected considering that the door-to-balloon time was longer in patients with COVID-19 suggestive symptoms than in patients without the symptoms (97.0 [74.8, 139.8] vs. 69.0 [55.0, 102.0] minutes, *P* < .001) during the same period (Table 1).These unexpected results can be explained by medical staff’s efforts to reduce the length of stay of the patients with COVID-19 related symptoms in the emergency room to minimize COVID-19 infection chance of other ED patients. Furthermore, even if a patient exhibited no symptoms suggestive of COVID-19, a COVID-19 test was required regardless to determine whether procedures should be conducted in isolation or general care. This led to an additional time taken for COVID-19 antigen testing, resulting in a longer door-to-balloon time compared to the pre-COVID-19 period. Some readers may think that statistically significant results could occur by chance when multiple statistical tests are conducted. However, as the pre-COVID-19 and COVID-19 periods are independent groups, there was no need for correction for multiple tests of significance.

 What can be confirmed in our study was that the door-to-balloon time increased for patients with symptoms suggestive of COVID-19 only during the COVID-19 period. This means that fever, difficulty breathing, and upper respiratory infection symptoms might be a bigger obstacle to early detection of STEMI patients in the COVID-19 period than in the pre-COVID-19 period. This difference can be explained by the changes that were implemented in the preparation process of equipment and personnel in the emergency room or cardiovascular laboratory during the COVID-19 period. In the COX proportional hazard simple regression, we found that the presence of symptoms suggestive of COVID-19 increased the 28-day mortality rate, but this effect was not statistically significant when other factors were included in the regression analysis. However, the presence of symptoms suggestive of COVID-19 had a *P* value of.0538, which was close to 0.05, indicating that it might be worth analyzing with a larger sample size in further studies. Additionally, it was found that the increase in door-to-balloon time for patients with COVID-19 suggestive symptoms did not have a statistically significant effect on 28-day mortality rate in STEMI patients, which is consistent with several previous studies showing that door-to-balloon time does not affect mortality of STEMI patients (Table 3).^28,29^

 It is worth reminding the fact that an increase in door-to-balloon time exacerbates ischemia in myocardium, from which occurrences of complications such as congestive heart failure are likely to increase.^30^ Therefore, shorter door-to-balloon time is important for patient’s prognosis and quality of life.

 There were cases where no isolation rooms were available at the emergency room when a patient suspected of AMI showed symptoms calling for isolation, incapacitating prompt treatment, and ambulances had to wait or re-transfer. Eventually, problems related to door-to-balloon time such as patient factors, as with patients’ reluctance to come to the hospital during the COVID-19 period, and systematic changes implemented in the emergency medical system in accommodations for COVID-19 situations have a combined effect on the increase in total ischemic time.^31^ Such situation was unavoidable because it is difficult to accurately diagnose AMI at the pre-hospital level. There was no established system that could provide information of availabilities of isolation rooms at different emergency rooms or of resource information of hospitals that were capable of definite treatment of time dependent patients such as that of AMI.

 In addition, we were able to confirm that the 28-day mortality rate of STEMI patients during the COVID-19 pandemic increased compared to that during the pre-COVID-19 period. Excluding death due to complications, cumulative hazard was analyzed for 28-day mortality rate for patients who died of STEMI. There was no difference in the hazard ratio correlated to the presence of symptoms suggestive of COVID-19 during the pre-COVID-19 period (2.5076, *P* = .0786). During the COVID-19 period, the hazard ratio was 2.7603 (*P* = .0245) for patients with the symptoms. However, Figure 3 could mislead as the cumulative incidence is estimated for times with few subjects in the risk set. Therefore, in future research, it will be imperative to increase the number of cases studied to minimize the potential for statistical errors. Since none of the STEMI patients in our study had COVID-19, it could be concluded that the cause of the increase in mortality was not due to the infectious disease itself, but an effect of the treatment process during COVID-19 period.

 Thus, scientific societies and health authorities should carefully determine critical symptoms that require isolation during the pandemic and implement it in medical institutions. Setting the range of isolation-requiring symptoms excessively broad will delay necessary treatment or quickly deplete medical resources such as the isolation rooms. However, if criteria of such symptoms are too focalized, given the increase in mortality among COVID-19-infected elderly patients with underlying diseases shown in many studies, infectious patients who may be overlooked for precautionary measures can infect other patients who may be at high risk of death and are routinely seen in emergency rooms or hospitals.

 This study has a limitation of having a retrospective design. There might be a difference in data fulfillment rate between the COVID-19 period and the pre-COVID-19 period. However, Korea currently manages all emergency room data with an integrated system titled the National Emergency Department Information System. All three institutions participating in this study recorded data fulfillment rates of more than 99%, minimizing the potential risk rate of type II errors. In addition, total ischemic time could not be analyzed because data on the time when patients’ symptoms started were not collected. However, accurate measurement of such time itself is not easy because there is limitation of relying only on patient’s memory for the timing. There is lack of aptitude in patients’ ability to distinguish symptoms such as between chest pain and abdominal pain.

## Conclusion

 In patients with STEMI during the COVID-19 period, the presence of symptoms suggestive of COVID-19 alone does not directly influence 28-day mortality. However, due to multifactorial reasons, patients who presented the symptoms showed a higher 28-day mortality rate than those who did not. Our study excluded effects made by characteristics of the disease itself and show that there was a difference in prognosis of STEMI patients correlated to the change of treatment process due to the presence of symptoms suggestive of COVID-19. Thus, health authorities should make careful decisions when designating symptoms that require isolation, based on advice of experts in various medical fields.

## Ethical issues

 The study was a retrospective study analyzing anonymized registry data of three hospitals using common EMR systems managed with integrated data. Therefore, formal approval from the ethical committee was deemed unnecessary. However, it was approved by Soonchunhyang University Seoul Hospital (data coordinating center) Institutional Review Board (SCHUH 2021-03-002).

## Competing interests

 Authors declare that they have no competing interests.

## Funding

 This work was supported by Soonchunhyang University Research Fund.

## Supplementary files

Supplementary file 1 contains Tables S1-S3.
