# Supplementary material for: Analysis of the Prognosis Outcomes and Treatment Delay Among ST-Segment Elevation Myocardial Infarction Patients in Emergency Department Based on the Presence of Symptoms Suggestive of COVID-19
Source: Int J Health Policy Manag. 2024 May 18;13:8207. doi: 10.34172/ijhpm.2024.8207 (PMC11270616; doi:10.34172/ijhpm.2024.8207)
Supplement: Supplementary file 1 — contains Tables S1-S3. [file ijhpm-13-8207-s001.pdf]

Article title: Analysis of the Prognosis Outcomes and Treatment Delay Among ST-Segment Elevation Myocardial Infarction Patients in Emergency Department Based on the Presence of Symptoms Suggestive of COVID-19

Journal name: International Journal of Health Policy and Management (IJHPM)

Authors' information: David Samuel Kwak[1], Joonbum Park[2]*

[1]Department of Family Medicine, Soonchunhyang University Seoul Hospital, Seoul, Republic of Korea.

[2]Department of Emergency Medicine, Soonchunhyang University Seoul Hospital, Seoul, Republic of Korea.

***Correspondence to:** Joonbum Park; Email: jesumania@gmail.com

Citation: Kwak DS, Park J. Analysis of the prognosis outcomes and treatment delay among ST-segment elevation myocardial infarction patients in emergency department based on the presence of symptoms suggestive of COVID-19. Int J Health Policy Manag. 2024;13:8207. doi:10.34172/ijhpm.2024.8207

Supplementary file 1

Table of content:

Table S1, p.2

Table S2, p.3

Table S3, p.4

Table S1. Characteristics of ST Segment Elevation Myocardial Infarction Patients Who Visited Emergency Departments During Pre-COVID-19 and COVID-19 Periods According to The Presence of Symptom Suggestive of COVID-19

Myocardial infarction	Symptoms suggestive of COVID-19 (+)		p-value	Symptoms suggestive of COVID-19 (-)		p-value
	Pre-COVID-19 (n=31)	COVID-19 (n=39)		Pre-COVID-19 (n=226)	COVID-19 (n=200)	
**Age**			>0.99			0.060
**18-64**	13 (41.9%)	16 (41.0%)		147 (65.0%)	112(56.0%)	
**≥65**	18 (58.1%)	23 (59.0%)		79 (35.0%)	88(44.0%)	
**Sex**			0.804			0.336
**Male**	21 (67.7%)	25(64.1%)		197 (87.2%)	167(83.5%)	
**Female**	10 (32.3%)	14(35.9%)		29 (12.8%)	33(16.5%)	
**COVID-19-related symptom**			0.053			
**Fever**	8 (25.8%)	5 (11.9%)		-	-	
**Dyspnea**	19 (61.3%)	30 (71.4%)		-	-	
**Other URI symptoms**	4 (12.9%)	7 (16.7%)		-	-	
**Vital signs**						
**Mean Arterial Pressure (mmHg)**	93.3 (81.0, 109.2)	93.3 (80.0, 112.8)	0.967	96.7 (83.3, 113.3)	98.5 (85.8, 113.3)	0.614
**Heart rate (/min)**	97.0 (87.0, 110.5)	89.0 (77.0, 100.5)	0.112	76.0 (65.0, 89.0)	78.5 (66.0, 93.0)	0.195
**Respiratory rate (/min)**	20.0 (18.0. 23.0)	20.0 (20.0, 26.0)	0.194	18.0 (18.0, 20.0)	18.0 (18.0, 20.0)	0.293
**Body temperature (℃)**	36.8 (36.1, 37.4)	36.5 (36.0, 36.9)	0.182	36.3 (36.0, 36.7)	36.2 (35.8, 36.5)	**0.036**
**SpO2 (%)**	95.0 (87.0, 97.5)	95.0 (85.0, 98.0)	0.802	98.0 (94.0, 99.0)	97.0 (97.0, 99.0)	**0.013**
**Interventions**						
**PCI treatment**	25 (80.7%)	34 (87.2%)	0.520	222 (98.2%)	186 (93.0%)	**0.008**
**Door to EKG time (min)**	8.0 (4.5, 11.5)	7.0 (5.0, 16.0)	0.590	6.0 (3.0, 9.0)	8.0 (4.0, 12.0)	**<0.001**
**Door-to-balloon time (min)**	73.0 (61.0, 92.0)	97.0 (74.8, 139.8)	**0.013**	67.0 (54.5, 80.0)	69.0 (55.0, 102.0)	0.2774

Abbreviations: COVID-19, coronavirus disease 2019; URI, upper respiratory syndrome; PCI, percutaneous coronary intervention; EKG, electrocardiogram.

Table S2. Comparing Outcomes of ST Segment Elevated Myocardial Infarction Patients Who Visited Emergency Departments According to The Presence of Symptom Suggestive of COVID-19

	Symptoms suggestive of COVID-19 (+)		p-value	Symptoms suggestive of COVID-19 (-)		p-value
	Pre-COVID-19 (n=31)	COVID-19 (n=39)		Pre-COVID-19 (n=226)	COVID-19 (n=200)	
**Length of ED stay**	144.0 (76.5, 269.0)	117.0 (80.0, 217.0)	0.958	108.0 (66.0, 163.0)	139.5 (83.5, 292.8)	**<0.001**
**ICU admission or Death in ED**	25 (80.7%)	32 (82.1%)	>0.99	214 (94.7%)	174 (87.0%)	**0.006**
**Admission days**	8.0 (3.0, 13.0)	6.0 (3.0, 9.5)	0.581	5.0 (3.0, 7.0)	4.0 (3.0, 7.0)	0.231
**Mortality within 28 days**	4 (15.4%)	8 (21.1%)	0.747	15 (6.8%)	13 (6.7%)	>0.99

Abbreviations: COVID-19, coronavirus disease 2019; ED, emergency department; ICU, intensive care unit

Table S3. Comparing Outcomes of Non-ST Segment Elevated Myocardial Infarction Patients Who Visited Emergency Departments During Pre-COVID-19 and COVID-19 Periods According to The Presence of Symptom Suggestive of COVID-19

	Pre-COVID-19 (n=283)		p-value	COVID-19 (n=204)		p-value
	Symptoms suggestive of COVID-19			Symptoms suggestive of COVID-19		
	Yes (n=94)	No (n=189)		Yes (n=62)	No (n=142)	
**EKG time (min)**	10.0 (7.0, 16.0)	8.0 (4.0, 13.0)	**0.0033**	12.5 (8.0, 17.8)	10.0 (7.0, 14.0)	**0.0078**
**Door-to-balloon time (min)**	177.0 (145.3, 243.0)	168.5 (115.3, 236.8)	0.4083	303.0 (218.3, 436.5)	207.0 (138.0, 320.5)	0.1619
**Length of ED stay**	243.5 (167.8, 324.8)	215.0 (155.0, 330.0)	0.2503	407.0 (277.8, 611.5)	342.5 (206.0, 653.0)	0.1561
**Admission days**	7.0 (4.0, 15.0)	4.0 (3.0, 6.0)	**<0.001**	8.0 (4.0, 12.0)	4.0 (3.0, 6.0)	**<0.001**
**Mortality within 28 days**	6 (7.1%)	5 (2.9%)	0.1847	9 (16.4%)	8 (6.1%)	0.0507

Abbreviations: COVID-19, coronavirus disease 2019; ED, emergency department
